# Healthcare resource utilisation in patients with exacerbations of COPD and associated cardiovascular events: the EXACOS-CV study

**DOI:** 10.1183/23120541.01212-2024

**Published:** 2025-11-03

**Authors:** Nathaniel M. Hawkins, Arshdeep K. Randhawa, Clementine Nordon, Manisha Talukdar, Suzanne McMullen, J. Paul Ekwaru, Tram Pham, Don D. Sin

**Affiliations:** 1 Centre for Cardiovascular Innovation, Division of Cardiology, University of British Columbia, Vancouver, BC, Canada; 2 AstraZeneca Canada Inc., Mississauga, ON, Canada; 3 Global Medical Evidence, AstraZeneca, Cambridge, UK; 4 Medlior Health Outcomes Research Ltd, Calgary, AB, Canada; 5 Department of Medicine (Division of Respirology), University of British Columbia, Vancouver, BC, Canada; 6 Centre for Heart Lung Innovation, St Paul's Hospital, Vancouver, BC, Canada

## Abstract

**Introduction:**

The EXACOS-CV Canada study described and evaluated treatment patterns, healthcare resource utilisation (HCRU), and costs in patients with COPD who experienced a severe cardiovascular (CV) event or death preceded or not preceded by an exacerbation.

**Methods:**

This longitudinal, retrospective cohort study included 142 787 individuals with COPD using administrative data from Alberta, Canada. The index event was a first hospitalisation for a CV event or all-cause death during follow-up. COPD-related treatment, HCRU and costs during the 12-month periods before and after the index events were described.

**Results:**

From the initial cohort, 43 564 (30.5%) patients with COPD experienced an index event; 15 100 (34.7%) had a COPD exacerbation in the 12 months prior. Patients whose index event was preceded (*versus* not preceded) by an exacerbation were dispensed higher amounts of COPD therapies in the 12 months prior (proportion of dual therapy: 36.9% *versus* 24.3%; proportion of triple therapy: 54.6% *versus* 16.3%) and 12 months following the CV event (proportion of dual therapy: 31.7% *versus* 27.4%; proportion of triple therapy: 53.4% *versus* 21.1%). Those with a preceding exacerbation also experienced higher rates of hospitalisations, emergency department admissions and general practitioner and specialist physician visits in both the 12 months prior to and following the index event.

**Discussion:**

Patients whose index events were preceded *versus* not preceded by an exacerbation received more treatment regimens and classes of COPD therapy but still incurred more HCRU. Despite the greater healthcare contacts, there remained large treatment gaps in this high-risk population.

## Shareable abstract

**Patients who experienced CV events after COPD exacerbations received more intensive COPD treatment and had more HCRU compared with patients whose CV events occurred without COPD exacerbations in the prior 12 months** https://bit.ly/4iuPxaW

## Introduction

Globally, COPD is a major cause of impaired quality of life, morbidity and mortality. In Canada, COPD affects approximately 12% of adults [1], is the most frequent cause of urgent hospitalisation and the fourth leading cause of death [2, 3]. COPD accounts for over CAD 1.5 billion in Canadian healthcare expenditures annually, with the majority attributable to management of COPD exacerbations [3]. The average annual cost per patient more than doubles in patients experiencing ≥2 compared with no exacerbations [4]; however, these costs may be significantly underestimated, as economic analyses have typically focused on COPD or respiratory-specific direct costs, including acute care, outpatient, outpatient consultations and prescriptions [5, 6].

Cardiovascular (CV) diseases are two- to five-fold more prevalent in individuals with *versus* without COPD [7]. Recent evidence demonstrates a consistent association between exacerbations and a broad range of major adverse CV events (MACEs), including myocardial infarction, stroke, heart failure and arrhythmia [8–10]. CV healthcare resource utilisation (HCRU) may therefore be a major unrecognised cost related to exacerbations, which to our knowledge has not previously been explored. This is particularly relevant as the 2023 Global Initiative for Chronic Obstructive Lung Disease (GOLD) report categorises patients with ≥2 moderate or ≥1 severe exacerbations per year in a single group termed “E” [11]. In this population, single inhaled triple therapy significantly reduced risk of exacerbations, hospitalisation and death compared with dual therapy in two large randomised controlled trials [12, 13]. In both studies the most common adjudicated cause of death was CV, and fewer CV deaths occurred in the triple-therapy group that contained inhaled corticosteroids (ICSs).

CV risk factors and disease cluster in patients with COPD, are modifiable and offer an opportunity to intervene and improve health outcomes. Comprehensively addressing “cardiopulmonary” risk would require a shift to multidisciplinary integrated care similar to cardiometabolic disease management in diabetes [14]. Understanding the economic impact of exacerbations followed by CV events may help justify earlier multidisciplinary interventions, trials testing new models of care, and health system change to enable earlier intensification of both cardiac and pulmonary therapies. The EXAcerbations of COPD and their OutcomeS in CardioVascular diseases (EXACOS-CV) study is examining the temporal relationship between exacerbations and CV events [15]. In Canada, the EXACOS-CV study demonstrated a 16-fold increased risk of the primary composite end-point of all-cause death or CV hospitalisation in the first 7 days following exacerbation [10].

In this analysis of the EXACOS-CV population, our objectives were to describe treatment patterns in a cohort of patients with COPD who experienced a severe CV event or all-cause death that was preceded or not preceded by an exacerbation, and to evaluate the economic impact of severe CV events preceded by an exacerbation.

## Methods

### Study design and data sources

This observational, longitudinal, retrospective cohort study is part of the multinational EXACOS-CV study, whose protocol has been published [15]. The EXACOS-CV Canada study examined patients in the province of Alberta, which provides universal access to hospitals, emergency departments (EDs), and outpatient physician services for free at the point of service for all 4.8 million registered residents of Alberta [16]. Research ethics board approval for this study was obtained from the Health Research Ethics Board of Alberta–Community Health Committee (HREBA.CHC-22-0006). A waiver of patient consent was granted due to the population-based, retrospective nature of the study.

De-identified linked data were obtained from six administrative databases (supplementary table S1).

### Population and follow-up

The Canadian EXACOS-CV cohort identified patients with incident or prevalent COPD who were ≥40 years old and residing in Alberta, Canada, between 1 April 2014 and 31 March 2020, using administrative data. To identify patients with COPD, this study relied on an algorithm used by Alberta Health based on International Classification of Diseases Ninth (Clinical Modification; ICD-9-CM) or Tenth (Canadian modification; ICD-10-CA) Revision codes for COPD occurring within a 2-year period: either 1) ≥2 ICD-9-CM codes in the primary position in the Physician Claims Dataset (physician billing); or 2) ≥1 ICD-10-CA codes in any position in the Discharge Abstract Database (inpatient hospitalisations) (supplementary table S2). The accuracy of identifying patients with COPD through an algorithm using ICD-10 diagnostic codes in the Canadian Institute of Health Information Discharge Abstract Database was validated by Gershon *et al.* [17]. Patients with a diagnosis of COPD before 1 April 2014, were categorised as prevalent and were assigned a cohort entry date (CED) of 1 April 2014. Patients who were diagnosed on or after 2 April 2014 were categorised as incident and were assigned a CED corresponding to their date of COPD diagnosis.

This analysis focused on the subpopulation who either died (all causes) or suffered ≥1 severe nonfatal CV event during follow-up. The index date was identified as the date of the first of these events. Severe CV events were defined as 1) hospitalisation ≥1 night (code I48 includes a visit to the ED) with any diagnosis code listed in supplementary table S3 as the “most responsible diagnosis” (reason for admission) or post-admission diagnosis (complication occurring during the hospital stay; diagnosis type “M” or “2” from supplementary table S4); and 2) not immediately followed by death (*i.e.* during the same hospitalisation), in which case the CV event was death, when the discharge disposition code was any of the codes in supplementary table S5.

### Study outcomes

**FIGURE 1:**
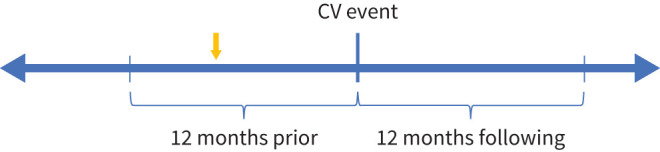
Time periods of study analysis. Yellow arrow identifies COPD exacerbation events. CV: cardiovascular.

COPD-related treatment patterns and HCRU were measured in the 12-month periods before and following the index date (figure 1). Follow-up lasted <12 months if the index event was death or if the patient otherwise died, moved out of province or due to administrative censoring (31 March 2020).

#### Treatment patterns

COPD-related treatment patterns were examined in the subgroups of patients with an index event and stratified by whether this event was or was not preceded by an exacerbation. A COPD exacerbation was classified per the Canadian Thoracic Society (CTS) guidelines as moderate (requiring a prescribed antibiotic and/or oral corticosteroid) or severe (requiring admission to hospital or ED visit) [18]. Treatment patterns were evaluated as proportions on COPD treatment regimens and annualised supply days (days per person-year) for monotherapies, dual therapies, triple therapies and other therapies for COPD. Monotherapy was defined as the dispensation of a single COPD-specific therapy during a given time period, while combination (dual or triple) therapy was defined as two or three (respectively) COPD-specific therapies dispensed at any time point during the same period. Categorisation of patients over the study period by treatment regimen was not mutually exclusive, as patients could switch treatment regimens. Treatment classes included ICS, short- and long-acting β-2 agonists (SABAs and LABAs), short- and long-acting muscarinic antagonists (SAMAs and LAMAs), oral corticosteroids, antibiotics, roflumilast, theophyllines, cardiac drugs and metabolic drugs. Monotherapy SABAs, and SAMAs are considered in the current guidelines as rescue therapy and monotherapy LABAs and LAMAs and combination treatment regimens are considered as maintenance therapy [11, 18].

#### HCRU and associated costs

HCRU was assessed in patients with an index event whether or not this event was preceded by an exacerbation in the past 12 months. HCRU was measured in the 12 months prior and the 12 months following the index date. All patients who were present for at least one day in the post-index period were counted. HCRU measures included COPD-specific hospitalisations, COPD-specific ED admissions and COPD-specific physician visits stratified by general practitioner (GP) and specialist physician (SP) type. Associated costs were assessed in the subgroup of patients who had a severe index event preceded by an exacerbation only. Costs are presented in 2021 Canadian dollars (CAD) and include total and mean annual costs of COPD-specific hospitalisations, ED admissions, GP and SP visits, and COPD- and cardiac-specific medication.

### Statistical analysis

All analyses were conducted using the SAS software v.9.4 (SAS Institute, Cary, North Carolina, USA). Descriptive statistics (frequency and mean±sd) were used to present cohort demographic characteristics, treatment regimens received prior to CED and COPD-specific HCRU and associated direct costs. No imputation of data was conducted.

Annualised rates of hospitalisations, ED admissions, GP and SP visits, and COPD- and cardiac-specific medications were calculated as 365×(number of HCRU)/(follow-up days). Annualised supply days were calculated as 365×(cumulative number of supply days)/(number of follow-up days within the 12-month period).

## Results

**FIGURE 2:**
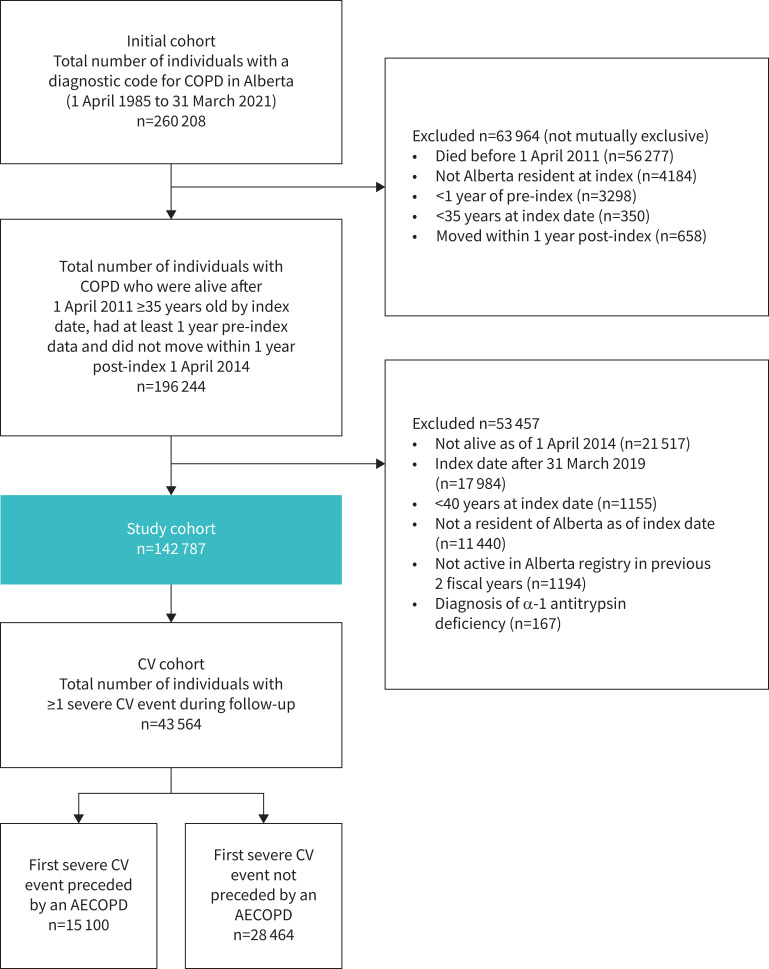
Cohort flow diagram. AECOPD: acute exacerbation of COPD; CV: cardiovascular.

The Canadian EXACOS-CV cohort included 142 787 patients with COPD, of whom 43 564 (30.5%) experienced ≥1 severe CV events or died during follow-up (figure 2). Of these, 34 068 patients (78.2%) died from CV-related, COPD-related, combined COPD/CV-related or other/unknown causes (supplementary table S6). An exacerbation occurred in the 12 months prior to the index event in 15 100 patients (34.7%). Baseline patient characteristics are presented in table 1 and for subgroups by experience of exacerbation in supplementary table S7.

**TABLE 1:** Baseline characteristics of the cohort who experienced a severe index event

Characteristic	Patients with an index event, n=43 564
**Age at CED, years**	75.0±11.5, 40.0–107.0
**Sex**	
Male	23 714 (54.4)
Female	19 850 (45.6)
**Residence at CED**	
Urban (Calgary, Edmonton)	26 260 (60.3)
Rural (Central, North, South)	17 304 (39.7)
**Neighbourhood income quintile at CED**	
1st	5437 (12.5)
2nd	7469 (17.1)
3rd	6079 (14.0)
4th	11 861 (27.2)
5th	12 718 (29.2)
**Prevalent patients (*versus* incident)**	31 660 (72.7)
**Number of exacerbations during the 12 months preceding CED**	
0	34 429 (79.0)
1	6575 (15.1)
2	1502 (3.4)
3	536 (1.2)
4+	522 (1.2)
**Comorbidities** ^#^	
Diabetes mellitus type 2	12 136 (27.9)
Dyslipidaemia^¶^	13 732 (31.5)
Ischaemic heart diseases	16 565 (38.0)
Hypertensive diseases	28 395 (65.2)
Heart failure	10 989 (25.2)
Cardiomyopathy	2136 (4.9)
Pulmonary oedema	929 (2.1)
Pulmonary hypertension	1976 (4.5)
Venous thromboembolism	6095 (14.0)
AF and other arrhythmias	11 554 (26.5)
Cerebrovascular disease	6696 (15.4)
Current (adult) asthma	1537 (3.5)
Chronic kidney disease, renal failure	8612 (19.8)
Severe mental illness^+^	2599 (6.0)
Anxiety disorder	7644 (17.5)
**Number of GP visits in the 12 months prior to CED**	17.4±20.7
**Medication dispensed in the 12 months prior to CED** ^§^	
Long-acting inhaled COPD drugs as single therapy^ ƒ^	15 490 (35.6)
Long-acting inhaled COPD drug as combination therapy^ ƒ^	15 701 (36.0)
Short-acting inhalers	16 467 (37.8)
Roflumilast and/or theophyllines	553 (1.3)
Cardiac medications	33 298 (76.4)
Metabolic medications	22 517 (51.7)

Data are presented as mean±sd, min–max or n (%). AF: atrial fibrillation; CED: cohort entry date; GP: general practitioner. ^#^: Comorbidities were based on all available look-back data except for “Current adult asthma” and “Anxiety”, which were based on 24-month look-back data. ^¶^: Includes hyperlipidaemia, hypercholesterolaemia and dyslipidaemia. ^+^: Severe mental illness includes recurrent and persistent depressive disorders, bipolar disorder and schizophrenia. ^§^: Medication dispense is defined as at least one dispensation during the 12 months prior to CED. ^ƒ^: Long-acting inhaled COPD drug use is defined as at least one dispensation during the period.

### Treatment patterns

**FIGURE 3:**
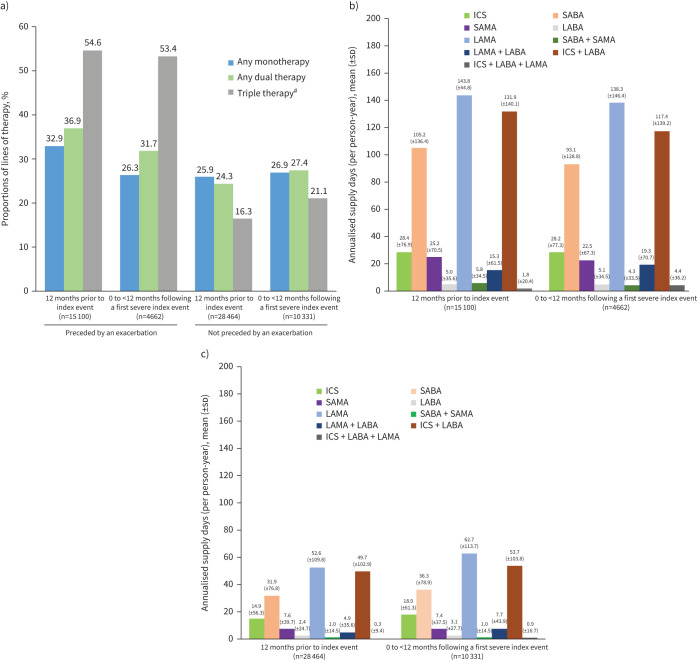
Proportions of patients on treatment regimens and mean annualised supply days for each treatment class among patients with an index event in the past 12 months preceded or not preceded by an exacerbation. a) Treatment regimens: index event preceded or not preceded by an exacerbation. b) Mean annualised supply days: index event preceded by an exacerbation. c) Mean annualised supply days: index event not preceded by an exacerbation. Patient numbers (x axis) reflect attrition during the 12 months post-index. ICS: inhaled corticosteroid; LABA: long-acting β-2 agonist; LAMA: long-acting muscarinic antagonist; SABA: short-acting β-2 agonist; SAMA: short-acting muscarinic antagonist. ^#^: Triple therapy consisted of an ICS, LABA and LAMA.

The proportion of patients on COPD treatment regimens and mean annualised supply days among patients with or without an exacerbation in the 12 months preceding the index event are presented in figure 3a–c and supplementary table S8.

Prior to the index event, the proportions of patients dispensed COPD monotherapy, dual or triple therapy were higher among those who experienced an exacerbation than those who did not (figure 3a and supplementary table S8). The greatest difference was among those dispensed triple therapy (54.6% *versus* 16.3%, respectively). Following the index event, the proportions of patients dispensed dual or triple therapy were higher among those whose index event was preceded *versus* not preceded by an exacerbation. The greatest difference was among those who were dispensed with triple therapy (53.4% *versus* 21.1%, respectively). The proportion of patients dispensed with monotherapy whose index event was preceded *versus* not preceded by an exacerbation was 26.3% *versus* 26.9%. However, patients with a pre-index exacerbation (*versus* not) had higher mean annualised supply days for monotherapies such as rescue medications (SABAs 105.2 days) and LAMAs (143.8 days) and lower supply days for maintenance triple-therapy medications (ICS+LABA+LAMA 1.8 days) (figure 3b,c).

Among patients with a pre-index exacerbation, the proportions of those dispensed COPD monotherapy, dual, or triple therapy were lower following *versus* prior to the index date (figure 3 and supplementary table S8), but no formal comparison was conducted.

### HCRU

**FIGURE 4:**
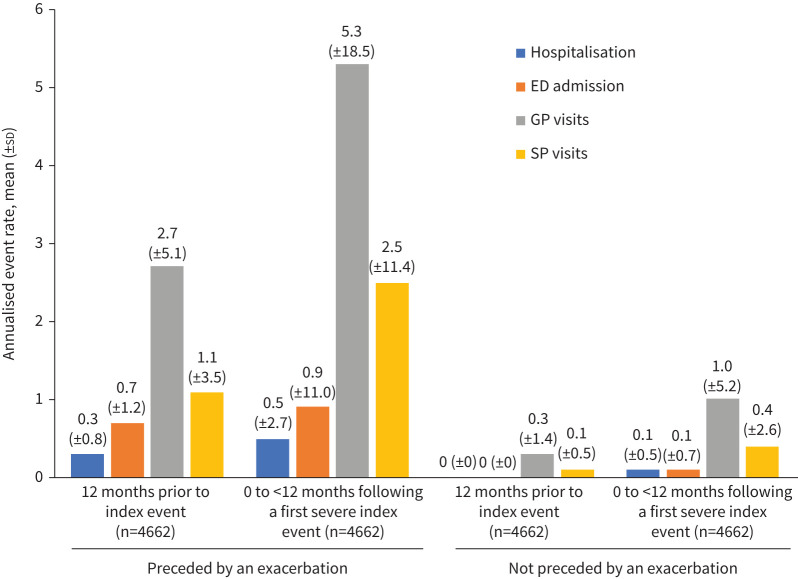
Mean±sd annualised healthcare resource utilisation (HCRU) rates among patients (survivors only) with an index event: COPD-specific hospitalisations^#^, emergency department (ED) admissions^#^ and/or general practitioner (GP)/specialist physician (SP) visits: preceded or not preceded by an exacerbation in the past 12 months. Patient number in the 12 months prior to index event represents survivors of the index event only. Annualised rates were calculated as (365×(number of HCRU)/(follow-up days)). ^#^: By definition, the numbers of COPD-specific hospitalisations and ED admissions during the 12 months prior to the index event among patients whose index event was not preceded by an exacerbation were 0.

Patients who experienced an exacerbation prior to their index event had higher mean annualised rates of COPD-specific HCRU, including hospitalisations, ED admissions, and GP and SP visits, compared with those who did not (figure 4 and supplementary table S9). All patients also had higher HCRU in the 12 months following *versus* prior to their index event, whether preceded or not preceded by an exacerbation.

### Costs

**FIGURE 5:**
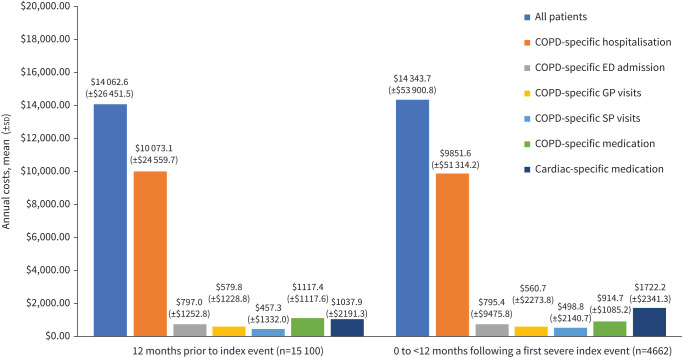
Mean annual costs among patients with an index event preceded by an exacerbation in the past 12 months. GP: general practitioner; ED: emergency department; SP: specialist physician.

Among all patients with an index event preceded by an exacerbation, mean total annual costs of all included healthcare categories were similar following *versus* prior to the index event (figure 5**;** supplementary table S10). The mean total annual costs of COPD-specific hospitalisation, ED admission, and GP and SP visits for all patients were similar following *versus* prior to the index event. The mean annual cost of COPD-specific medication was lower and the mean annual cost of cardiac-specific medication was higher following *versus* prior to the index event.

## Discussion

We report several key findings in this real-world study in patients with COPD and severe CV events. First, when preceded by an exacerbation, there was markedly increased HCRU compared with those without preceding exacerbation. Second, HCRU increased notably following severe CV events irrespective of exacerbation history. Third, most of the COPD-specific costs related to hospitalisation as opposed to primary care visits or medications. Finally, although patients with prior exacerbations did receive higher levels of guideline-directed medical therapy, significant treatment gaps remained, with only around half of patients prescribed inhaled triple therapy, a practice that is inconsistent with most major COPD guidelines [11].

A large amount of medications, both treatment regimens and drug classes, were dispensed to patients whether their index event was preceded or not preceded by an exacerbation. However, mean annualised supply days were higher among patients with a preceding exacerbation. Although higher dispensation of maintenance (ICS+LABA+LAMA) triple therapy was observed among patients with a preceding exacerbation, exacerbations were also associated with higher mean annualised supply days for rescue or inappropriate monotherapy inhalers (SABA and ICS) and dispensation of less-effective maintenance therapies (LAMA and ICS+LABA). This raises the possibility that some of these patients were relying on suboptimal maintenance therapy and rescue agents, which may lead to a cycle of undertreatment and a potentially higher rate of exacerbations, particularly among patients with no history of either an exacerbation or index event who are at increased risk of these events. A single-site analysis by Saint-Pierre [19] found that only 22 of 111 (20.0%) of patients in Ontario, Canada, who experienced an exacerbation and who were candidates for review of their inhaled therapy according to the CTS guidelines received the recommended optimisation.

GOLD 2023 recommends considering initiation of triple therapy in patients with previous moderate to severe exacerbations (GOLD group E), particularly those with elevated blood eosinophils [11]; however, it should be noted that patients’ blood eosinophil levels were not recorded in this study. The CTS recommends treatment “step up” to triple therapy for patients at risk of exacerbation despite the use of monotherapy or dual therapy. This recommendation is based on evidence demonstrating the benefits of triple therapy in patients with a high risk of exacerbation [12, 18, 20]. The CTS also suggests that triple therapy be used in patients taking LABA+LAMA with persistent dyspnoea and poor health status in the past year, noting that dyspnoea and exacerbation often manifest in the same patient [18].

Mean annual costs for all included healthcare categories in the 12 months following the index event were similar to those in the 12 months previous. These results may reflect the impact of post-CV mortality. The main drivers of total costs (aside from cardiac-specific medications) were COPD-specific hospitalisations and COPD-specific ED visits. These findings are consistent with existing Canadian data. A previous Canadian analysis found that the annual direct cost of COPD was approximately CAD$2000 per patient, with costs largely driven by unscheduled care visits, including primary care or SP contacts, inpatient stays and ED visits [21]. Between 2022 and 2023, COPD/bronchitis was identified as the second most frequent cause of hospitalisation in adults in Canada, behind only childbirth [22], and COPD-related hospitalisations have increased in Canada since 2010 even with adjustment for growth and ageing of the population [23]. Using the Canadian Institute for Health Information's Patient Cost Estimator, the estimated average cost of hospitalisation in Canada was CAD$8202 per patient [24]. The findings from the present study align with real-world studies in other regions, confirming that COPD with comorbid CV is associated with increased risk of mortality, hospitalisation, longer hospital stay and ED visits compared with patients with COPD alone [25–30].

The high HCRU observed in patients with COPD and severe CV events, particularly with exacerbations, should be considered both a target to improve outcomes and reduce costs and an opportunity to enact that improvement. We identified high use of monotherapies and less-effective maintenance therapies among patients with prior exacerbations, in conjunction with frequent healthcare contacts for respiratory reasons. Each of these contact points is an opportunity to optimise both inhaled and other guideline-directed respiratory and CV therapy. An analysis from Canadian primary care demonstrated clustering of traditional CV risk factors in patients with COPD, infrequent monitoring, suboptimal control and underuse of simple therapies such as angiotensin-converting enzyme inhibitors and statins [31]. Clinicians, health system administrators and policy makers should be aware of this heightened cardiopulmonary risk and take steps to intensify therapy following exacerbations, particularly after subsequent CV events.

### Strengths and limitations

The strengths of this study include the use of large population-based administrative health data comprising hospital, ambulatory, pharmacy-level prescription claims and vital statistics data. Patients were treated within a single-payer health insurance system, which ensured that all patients had identical coverage that can be linked across these administrative health databases and minimised the risk of missing an exposure (outpatient visit or hospitalisation for COPD), or an outcome (hospitalisation for index event or death). The use of administrative health databases minimised selection bias due to patients’ employment or socioeconomic status and enabled a large cohort of patients to be identified who have both COPD and an index event, giving sufficient statistical power to conduct our analyses. A minimum of 2 years pre-enrolment data to assess patient characteristics and identify “true” incident cases followed by up to 6 years of follow-up enabled a robust estimate of the risk of index events following subsequent exacerbations.

This study had several limitations. First, administrative health data that are not collected specifically for research purposes may affect the accuracy and reporting of these variables that are subject to limitations with respect to missing or incorrectly coded data. Specifically, hospital medication dispenses were not captured in the Pharmaceutical Information Network dataset. Second, to improve the specificity of our algorithm and minimise the risk of considering non-exacerbation events as moderate exacerbations, we considered only the most severe of the exacerbations managed in the outpatient setting, which could lower the generalisability of our findings. Smoking status was not adequately collected in the database and was therefore not assessed. This was listed as a limitation in other EXACOS-CV studies [10, 32–35], as well as in similar studies in Canada [23] and elsewhere [29]. Use of spirometry was not included in patient classification or analysis in line with similar studies [10, 23, 29, 32–35]. Finally, the complexity inherent to the CV outcomes we assessed, and the range of possible symptomatology and treatment approaches may have affected estimates of patients’ particular outcomes.

### Conclusions

Our findings underscore the high COPD-specific HCRU and costs associated with severe CV events in patients with COPD who have experienced exacerbations in the 12 months prior to index events. Overall, these results highlight opportunities for more effective patient management to reduce HCRU and costs associated with severe CV events in patients with COPD. While appropriate treatment and intensified patient monitoring following exacerbations of any severity have potential to reduce the risk of severe CV events, optimising interventions after an event may also help reduce HCRU and costs.

## Data Availability

The dataset supporting the conclusions of this article was derived from Alberta Health administrative data. De-identified data were released to Medlior Health Outcomes Research by Alberta Health following ethical approval and a data request. Data from this study are not publicly available and cannot be shared for privacy reasons and ethical restrictions as per the research agreement with Alberta Health.

## Supplementary material

**Please note:** supplementary material is not edited by the Editorial Office, and is uploaded as it has been supplied by the author. Supplementary material 01212-2024.SUPPLEMENT
