# Supplementary material for: Healthcare resource utilisation in patients with exacerbations of COPD and associated cardiovascular events: the EXACOS-CV study
Source: ERJ Open Res. 2025 Nov 3;11(6):01212-2024. doi: 10.1183/23120541.01212-2024 (PMC12581169; doi:10.1183/23120541.01212-2024)
Supplement: Supplementary file 1 [file 01212-2024.SUPPLEMENT.pdf]

Supplementary Material

Supplementary table S1. Administrative databases from which data were collected

National Ambulatory Care Reporting System	Captures all visits to the 93 EDs in Alberta and contains dates, disposition and up to 10 physician-assigned diagnoses per visit
Alberta Population Registry	Captures basic demographic information, including age, gender, and geographic zone
Vital Statistics - deaths	Captures date of death
Discharge Abstract Database	Captures all acute care hospitalisations and includes admission and discharge dates, elective vs non-elective, the primary diagnosis and up to 24 other diagnoses assigned by the attending physician
Pharmaceutical Information Network	Captures all prescriptions dispensed in Alberta pharmacies and is not restricted by age
Practitioner Claims database	Captures all outpatient physician visits with up to 3 diagnoses per visit

ED, emergency department

Supplementary table S2. ICD-9-CM/ICD-10-CA codes related to COPD

ICD-9-CM / ICD-10-CA	Code	Code description	Definition algorithm during the cohort year
ICD-9-CM	491	Chronic bronchitis	≥ 2 outpatient visits (physician claims) in the primary position on separate days and within 2 years (2nd visit as index date)
	492	Emphysema	
	496	Chronic airways obstruction, not elsewhere classified	
ICD-10-CA	J41	Chronic bronchitis	1 inpatient (DAD) admission in any position (discharge date as index date)
	J42	Unspecified chronic bronchitis	
	J43	Emphysema	
	J44	Other chronic obstructive pulmonary disease	

DAD, Discharge Abstract Database; ICD-9-CM, International Classification of Diseases, Ninth Revision, Clinical Modification; ICD-10-CA, International Statistical Classification of Diseases and Related Health Problems, Tenth Revision, Canadian Modification.

Supplementary table S3. List of non-fatal severe index events

Categories of non-fatal severe index event	ICD-10 codes
ACS: Acute myocardial infarction and unstable angina	• I21 Acute myocardial infarction or<br>• I20.0 Unstable angina
HF decompensation	• I50 (Congestive) HF including<br>• J81 acute pulmonary edema
Cerebral ischemia: Cerebral infarction; and TIA	• I63 Cerebral infarction or<br>• G45 TIA (“Transient cerebral ischemic attacks and related syndromes”)
Arrhythmias: Newly diagnosed AF; and other serious cardiac arrhythmia including cardiac arrest	• I48 AF and flutter or<br>• I49 Other cardiac arrhythmias or<br>• I46 Cardiac arrest

ACS, acute coronary syndrome; AF, atrial fibrillation; AMI, acute myocardial infarction; CV, cardiovascular; HF, heart failure; ICD-10, International Classification of Diseases, Tenth Revision, Canadian modification; TIA, transient ischemic attack.

Supplementary table S4. Diagnosis Type in the Discharge Abstract Database (DAD)

Code	Type explanation
M	Most responsible diagnosis
1	Comorbidity – preadmit
2	Comorbidity – post admit
3	Secondary diagnosis
5	Admitting diagnosis
6	Proxy most responsible diagnosis
W, X, Y	Service transfer diagnosis
9	External cause of injury codes
0	Newborn discharge abstracts

Supplementary table S5. List of death-related discharge disposition codes

Applicable fiscal years	Disposition code	Description
FY2010 - FY2017	07	Died
FY2018 - Present	66	Died while on pass/leave
	67	Suicide out of facility
	71	Dead on arrival
	72	Died in facility
	73	Medical assistance in dying
	74	Suicide in facility

Supplementary table S6. Number and causes of death

Cause of death	Estimate
Number of patients, n	43,564
Death from any cause, n (%)	34,068 (78.2)
COPD-related death, n (%)	4,973 (11.4)
CV-related death, n (%)	10,132 (23.3)
Death due to both COPD and CV, n (%)	81 (0.2)
Death due to other cause, n (%)	18,882 (43.3)

Supplementary table S7. Baseline characteristics by timing of CV event

Characteristic	CV patients who had no exacerbations during follow-up (n=21,250)	CV patients who experienced exacerbations during follow-up (n=22,314)
**Age at CED, years**		
Mean (SD), min-max	75.7 (12.0), 67.0 - 85.0	74.2 (11.0), 67.0 - 83.0
**Sex, n (%)**		
Male	11,587 (54.5)	12,127 (54.3)
Female	9,663 (45.5)	10187 (45.7)
**Residence at CED, n (%)**		
Urban (Calgary, Edmonton)	13,185 (62.0)	13,075 (58.6)
Rural (Central, North, South)	8,065 (38.0)	9,239 (41.4)
**Neighborhood income quintile at CED, n (%)**		
1st	2,815 (13.2)	2,622 (11.8)
2nd	3,814 (17.9)	3,655 (16.4)
3rd	2,962 (13.9)	3,117 (14.0)
4th	5,513 (25.9)	6,348 (28.4)
5th	6,146 (28.9)	6,572 (29.5)
**Prevalent patients (vs. incident), n (%)**	15,217 (71.6)	16,443 (73.7)
**Number of exacerbations during the 12 months preceding CED, n (%)**		
0	19,006 (89.4)	15,423 (69.1)
1	2,009 (9.5)	4,566 (20.5)
2	184 (0.9)	1,318 (5.9)
3	32 (0.2)	504 (2.3)
4+	19 (0.1)	503 (2.3)
**Comorbidities, n (%)**[a]		
Diabetes mellitus type 2	6,093 (28.7)	6,043 (27.1)

Dyslipidemia[b]	6,784 (31.9)	6,948 (31.1)
Ischemic heart diseases	8,084 (38.0)	8,481 (38.0)
Hypertensive diseases	13,971 (65.7)	14,424 (64.6)
Heart failure	5,051 (23.8)	5,938 (26.6)
Cardiomyopathy	1,017 (4.8)	1,119 (5.0)
Pulmonary edema	423 (2.0)	506 (2.3)
Pulmonary hypertension	813 (3.8)	1,163 (5.2)
Venous thromboembolism	2,818 (13.3)	3,277 (14.7)
AF and other arrhythmias	5,720 (26.9)	5,834 (26.1)
Cerebrovascular disease	3,541 (16.7)	3,155 (14.1)
Current (adult) asthma	540 (2.5)	997 (4.5)
Chronic kidney disease, renal failure	4,269 (20.1)	4,343 (19.5)
Severe mental illness[c]	1,236 (5.8)	1,363 (6.1)
Anxiety disorder	3,561 (16.8)	4,083 (18.3)
**Number of GP visits in the 12 months prior to CED**		
Mean (SD)	18.3 (22.9)	16.5 (18.3)
**Medication dispensed in the 12 months prior to CED, n (%)[d]**		
Long-acting inhaled COPD drugs as single therapy	4,486 (21.1)	11,004 (49.3)
Long-acting inhaled COPD drugs as combination therapy[e]	4,535 (21.3)	11,166 (50.0)
Short-acting inhalers	5,190 (24.4)	11,277 (50.5)
Roflumilast and/or theophylline, n (%)	68 (0.3)	485 (2.2)
Cardiac medications	15,931 (75.0)	17,367 (77.8)
Metabolic medications	10,888 (51.2)	11,629 (52.1)

[a] Comorbidities were based on all available look-back data except for ‘Current adult asthma’ and ‘Anxiety’ which were based on 24-month look-back data.
[b] Includes hyperlipidemia, hypercholesterolemia, dyslipidemia. [c] Severe mental illness includes recurrent and persistent depressive disorders, bipolar disorder and schizophrenia. [d] Medication dispense is defined as at least 1 dispensation during the 12-months prior to CED. [e] Long-acting inhaled COPD drug use is defined as at least one dispensation during the period.

AF, atrial fibrillation; CED, cohort entry date; GP, general practitioner; IQR, interquartile range; SD, standard deviation.

Supplementary table S8. COPD medications reported by time period whose first severe index event was preceded vs. not preceded by an exacerbation

	Patient Group									
	First severe index event preceded by an exacerbation					First severe index event not preceded by an exacerbation				
	12 months prior to index event N=15,100	0-<1 month following index event N=4,662	1-<3 months following index event N=4,514	3-<12 months following index event N=4,187	0-<12 months following index event N=4,662	12 months prior to index event N=28,464	0-<1 month following index event N=10,331	1-<3 months following index event N=10,027	3-<12 months following index event N=9,483	0-<12 months following index event N=10,331
**Proportion on treatment regimen, n (%)**										
**Monotherapies**										
Any monotherapy	4,974 (32.9)	902 (19.3)	875 (19.4)	905 (21.6)	1,227 (26.3)	7,383 (25.9)	2,096 (20.3)	2,126 (21.2)	2,249 (23.7)	2,780 (26.9)
ICS	246 (1.6)	29 (0.6)	34 (0.8)	39 (0.9)	52 (1.1)	595 (2.1)	138 (1.3)	142 (1.4)	177 (1.9)	221 (2.1)
SABA or SAMA	1,998 (13.2)	290 (6.2)	263 (5.8)	311 (7.4)	451 (9.7)	3,572 (12.5)	895 (8.7)	876 (8.7)	938 (9.9)	1,233 (11.9)
SABA	1,785 (11.8)	268 (5.7)	239 (5.3)	275 (6.6)	406 (8.7)	3,149 (11.1)	810 (7.8)	787 (7.8)	849 (9.0)	1,107 (10.7)
SAMA	268 (1.8)	22 (0.5)	25 (0.6)	39 (0.9)	50 (1.1)	480 (1.7)	88 (0.9)	90 (0.9)	100 (1.1)	145 (1.4)
LABA or LAMA	3,206 (21.2)	605 (13.0)	598 (13.2)	600 (14.3)	817 (17.5)	3,633 (12.8)	1,101 (10.7)	1,143 (11.4)	1,219 (12.9)	1,489 (14.4)
LABA	119 (0.8)	19 (0.4)	19 (0.4)	25 (0.6)	32 (0.7)	147 (0.5)	44 (0.4)	45 (0.4)	47 (0.5)	62 (0.6)
LAMA	3,095 (20.5)	586 (12.6)	579 (12.8)	577 (13.8)	790 (16.9)	3,491 (12.3)	1,057 (10.2)	1,098 (11.0)	1,176 (12.4)	1,431 (13.9)
**Dual therapies**										
Any dual therapy, n (%)	5,579 (36.9)	1,155 (24.8)	1,085 (24.0)	1,142 (27.2)	1,477 (31.7)	6,927 (24.3)	2,355 (22.8)	2,283 (22.8)	2,284 (24.1)	2,831 (27.4)
SABA+SAMA	710 (4.7)	94 (2.0)	88 (1.9)	96 (2.3)	146 (3.1)	743 (2.6)	141 (1.4)	160 (1.6)	169 (1.8)	247 (2.4)
LABA+LAMA	919 (6.1)	206 (4.4)	213 (4.7)	239 (5.7)	290 (6.2)	784 (2.8)	275 (2.7)	297 (3.0)	363 (3.8)	418 (4.0)
ICS+LABA	3,367 (22.3)	669 (14.4)	615 (13.6)	632(15.1)	845(18.1)	4,531 (15.9)	1,544 (14.9)	1,440 (14.4)	1,398 (14.7)	1,749 (16.9)
ICS+LAMA	473 (3.1)	105 (2.3)	98 (2.2)	114 (2.7)	147 (3.2)	370 (1.3)	137 (1.3)	141 (1.4)	150 (1.6)	190 (1.8)
ICS+SABA	494 (3.3)	94 (2.0)	82 (1.8)	85 (2.0)	119 (2.6)	714 (2.5)	272 (2.6)	249 (2.5)	255 (2.7)	321 (3.1)
ICS+SAMA	45 (0.3)	<10	<10	<10	10 (0.2)	74 (0.3)	11 (0.1)	10 (0.1)	13 (0.1)	19 (0.2)

**Triple therapies**										
ICS+LABA+LAMA, n (%)	8,240 (54.6)	2,297 (49.3)	2,235 (49.5)	2,098 (50.1)	2,490 (53.4)	4,642 (16.3)	1,834 (17.8)	1,885 (18.8)	1,905 (20.1)	2,183 (21.1)
**Annualised supply days (per person-year), mean (SD)**										
**Monotherapies**										
ICS	28.4 (76.9)	31.8 (102.0)	27.3 (88.4)	27.0 (77.5)	28.2 (77.3)	14.9 (56.3)	19.4 (82.0)	18.5 (73.7)	16.9 (61.3)	18.0 (61.3)
SABA	105.2 (136.4)	123.0 (177.6)	95.6 (151.4)	84.4 (129.5)	93.1 (128.8)	31.9 (76.8)	48.7 (117.1)	39.1 (97.4)	32.8 (78.8)	36.3 (78.9)
SAMA	25.2 (70.5)	28.5 (92.0)	23.3 (80.8)	20.1 (67.3)	22.5 (67.3)	7.6 (39.7)	9.4 (53.3)	8.3 (46.8)	6.9 (37.6)	7.4 (37.5)
LABA	5.0 (35.6)	6.0 (45.9)	5.2 (38.5)	5.0 (34.7)	5.1 (34.5)	2.4 (24.7)	3.3 (33.1)	3.4 (32.7)	3.0 (28.6)	3.1 (27.7)
LAMA	143.8 (44.8)	154.6 (182.6)	146.5 (169.8)	131.8 (148.6)	138.3 (146.4)	52.6 (109.8)	66.5 (137.3)	66.4 (132.2)	61.4 (116.4)	62.7 (113.7)
**Dual therapies**										
SABA+SAMA	5.8 (34.5)	4.9 (42.2)	3.9 (31.6)	3.3 (26.5)	4.3 (33.5)	1.0 (14.5)	1.1 (18.0)	1.0 (16.1)	0.7 (12.9)	1.0 (14.5)
LABA+LAMA	15.3 (61.5)	19.2 (81.8)	17.5 (74.2)	19.4 (72.1)	19.3 (70.7)	4.9 (35.6)	6.5 (47.1)	6.5 (45.0)	7.9 (46.5)	7.7 (43.9)
ICS+LABA	131.9 (140.1)	133.1 (178.6)	118.7 (162.1)	112.9 (140.0)	117.4 (139.2)	49.7 (102.9)	58.9 (133.0)	55.7 (121.2)	52.8 (105.6)	53.7 (103.8)
**Triple therapies**										
ICS+LABA+LAMA	1.8 (20.4)	3.4 (35.7)	3.4 (34.3)	3.9 (34.2)	4.4 (36.2)	0.3 (9.4)	0.7 (16.6)	0.7 (15.2)	0.8 (15.2)	0.9 (16.7)

ICS, inhaled corticosteroid; LABA, long-acting beta agonist; LAMA, long-acting muscarinic antagonist; SABA, short-acting beta-2 agonist; SAMA, short-acting muscarinic antagonist; SD, standard deviation.

Supplementary table S9. Rates of HCRU among patients whose first severe index event was preceded vs. not preceded by an exacerbation in the past 12 months

	Patient Group									
	First severe index event preceded by an exacerbation					First severe index event not preceded by an exacerbation				
	12 months prior to index event N=4,662	0-<1 month following index event N=4,662	1-<3 months following index event N=4,514	3-<12 months following index event N=4,187	0-<12 months following index event N=4,662	12 months prior to index event N=10,331	0-<1 month following index event N=10,331	1-<3 months following index event N=10,027	3-<12 months following index event N=9,483	0-<12 months following index event N=10,331
**Hospitalisations (COPD-specific)**										
Total number of hospitalisations, sum	1,564	400	194	751	1,345	0	51	100	351	502
Mean (SD) annualised hospitalisation rate	0.3 (0.8)	1.2 (4.4)	0.3 (1.8)	0.3 (1.3)	0.5 (2.7)	0 (0)	0.1 (0.9)	0.1 (0.7)	0.1 (0.6)	0.1 (0.5)
Number of patients with ≥1 hospitalisation, n (%)	1101 (23.6)	381 (8.2)	173 (3.8)	522 (12.4)	920 (19.7)	0 (0)	50 (0.5)	97 (1.0)	283 (3.0)	410 (4.0)
Mean (SD) annualised rate among patients with ≥1 hospitalisation	1.4 (1.1)	14.1 (7.0)	7.9 (5.0)	2.6 (2.7)	2.6 (5.6)	0 (0)	13.0 (2.0)	6.7 (2.0)	2.4 (2.8)	1.7 (1.9)
**ED admissions (COPD-specific)**										
Total number of admissions, sum	3,219	611	387	1,277	2,275	0	68	158	593	819
Mean (SD) annualised admission rate	0.7 (1.2)	1.9 (11.8)	0.6 (2.6)	0.5 (2.0)	0.9 (11.0)	0 (0)	0.1 (1.1)	0.1 (2.1)	0.1 (0.6)	0.1 (0.7)
Number of patients with ≥1 admission, n (%)	1966 (42.2)	559 (12.0)	296 (6.6)	743 (17.7)	1286 (27.6)	0 (0)	64 (0.6)	143 (1.4)	441 (4.6)	607 (5.9)
Mean (SD) annualised rate among patients with ≥1 admission	1.7 (1.4)	15.6 (30.9)	8.8 (5.7)	3.0 (4.0)	3.4 (20.8)	0 (0)	13.8 (3.9)	8.5 (15.1)	2.2 (1.9)	1.9 (2.3)

**GP visits (COPD-specific)**										
Total number of admissions, sum	12,222	3,207	2,796	7,830	13,833	3,471	997	1,175	4,645	6,817
Mean (SD) annualised admission rate	2.7 (5.1)	9.1 (30.5)	4.3 (17.3)	3.5 (12.5)	5.3 (18.5)	0.3 (1.4)	1.3 (8.5)	0.8 (5.3)	0.8 (5.5)	1.0 (5.2)
Number of patients with ≥1 admission, n (%)	2997 (64.3)	982 (21.1)	936 (20.7)	1748 (41.7)	2410 (51.7)	1757 (17.0)	488 (4.7)	605 (6.0)	1581 (16.7)	2160 (20.9)
Mean (SD) annualised rate among patients with ≥1 admission	4.1 (5.9)	43.4 (54.3)	20.6 (33.3)	8.5 (18.3)	10.2 (24.7)	2.0 (3.0)	26.7 (29.2)	13.3 (17.4)	5.1 (12.8)	4.6 (10.5)
**SP visits (COPD-specific)**										
Total number of admissions, sum	4,968	2,479	1,166	2,895	6,540	1,226	915	592	1,497	3,004
Mean (SD) annualised rate	1.1 (3.5)	6.9 (25.8)	1.7 (10.0)	1.2 (5.5)	2.5 (11.4)	0.1 (0.5)	1.1 (8.1)	0.4 (4.5)	0.3 (1.6)	0.4 (2.6)
Number of patients with ≥1 SP visit, n (%)	1309 (28.1)	684 (14.7)	479 (10.6)	887 (21.2)	1453 (31.2)	787 (7.6)	385 (3.7)	331 (3.3)	748 (7.9)	1172 (11.3)
Mean (SD) annualised rate among patients with ≥1 SP visit	3.9 (5.8)	47.4 (51.1)	16.0 (26.6)	5.7 (11.0)	8.0 (19.3)	1.6 (1.1)	30.2 (29.4)	12.5 (21.6)	3.2 (4.8)	3.7 (7.1)

COPD, chronic obstructive pulmonary disease; GP, general practitioner; ED, emergency department; HCRU, healthcare resource utilisation; SD, standard deviation; SP, specialist physician.

Supplementary table S10. Cost burden among patients with COPD and an index event (including death)

	Time Period				
	12 months prior to index event N=15,100	0 - <1 month following the first severe index event N =4,662	1 - <3 months following a first severe index event N =4,517	3 - <12 months following a first severe index event N =4,193	0 - <12 months following a first severe index event N=4,662
**Total cost of COPD-specific hospitalisations**					
Total cost, sum	$149,603,453	$10,963,219.8	$5,648,322	$10,554,346.4	$27,165,888.1
Mean (SD) annual costs	$10,073.1 ($24,559.7)	$30,545.1 ($163,308.0)	$8,671.4 ($67,212.7)	$5,133.0 ($28,017.2)	$9,851.6 ($51,314.2)
Number of patients with positive[a] total cost, n (%)	6,099 (40.4)	413 (8.9)	253 (5.6)	556 (13.3)	946 (20.3)
Mean (SD) annual costs in patients with positive[a] total cost	$24,939.1 ($33,506.6)	$344,797.4 ($439,429.7)	$154,714.2 ($241,286.0)	$38,724.3 ($68,029.3)	$48,549.7 ($105,388.1)
**Total cost of COPD-specific ED admissions**					
Total cost, sum	$11,836,191.8	$584,084.7	$290,233.6	$994,334.2	$1,868,652.5
Mean (SD) annual costs	$797.0 ($1,252.8)	$1,765.5 ($10,368.4)	$441.2 ($2,115.0)	$417.8 ($1,500.4)	$795.4 ($9,475.8)
Number of patients with positive[a] total cost, n (%)	7,752 (51.3)	558 (12.0)	295 (6.5)	741 (17.7)	1282 (27.5)
Mean (SD) annual costs in patients with positive[a] total cost	$1,552.4 ($1,372.9)	$14,750.1 ($26,603.2)	$6,750.8 ($5,091.7)	$2,361.0 ($2,853.3)	$2,892.4 ($17,906.5)
**Total cost of COPD-specific GP visits**					
Total cost, sum	$8,611,641.6	$371,827.1	$268,290.7	$733,413	$1,373,530.7
Mean (SD) annual costs	$579.8 ($1,228.8)	$1,068.1 ($3,603.1)	$414.2 ($1,811.5)	$339.2 ($1,357.7)	$560.7 ($2,273.8)
Number of patients with positive[a] total cost, n (%)	10,662 (70.6)	982 (21.1)	936 (20.7)	1,748 (41.7)	2410 (51.7)
Mean (SD) annual costs in patients with positive[a] total cost	$821.2 ($1,392.9)	$5,070.7 ($6,431.7)	$1,997.7 ($3,559.8)	$813.1 ($2,008.4)	$1,084.6 ($3,071.6)
**Total cost of COPD-specific SP visits**					
Total cost, sum	$6,791,674.7	$533,156.2	$244,458.2	$578,956.1	$1,356,570.5
Mean (SD) annual costs	$4,57.3 ($1,332.0)	$1,473.5 ($5,303.7)	$356.4 ($2,159.1)	$246.4 ($1,278.3)	$498.8 ($2,140.7)
Number of patients with positive[a] total cost, n (%)	5268 (34.9)	684 (14.7)	479 (10.6)	887 (21.2)	1,453 (31.2)

Mean (SD) annual costs in patients with positive[a] total cost	$1,310.8 ($1,991.8)	$10,042.9 ($10,284.5)	$3,359.0 ($5,822.8)	$1,163.2 ($2,579.4)	$1,600.5 ($3,598.0)
**Total cost of COPD-specific medication**					
Total cost, sum	$16,596,038.1	$373,218	$621,111.3	$2,493,919.7	$3,488,248.9
Mean (SD) annual costs	$1,117.4 ($1,117.6)	$1,021.1 ($1,918.2)	$878.8 ($1,390.6)	$910.5 ($1,125.8)	$914.7 ($1,085.2)
Number of patients with positive[a] total cost, n (%)	13,590 (90.0)	2,112 (45.3)	2,464 (54.5)	3,232 (77.1)	3,847 (82.5)
Mean (SD) annual costs in patients with positive[a] total cost	$1,241.6 ($1,110.7)	$2,254.0 ($2,311.7)	$1,610.0 ($1,538.1)	$1,179.5 ($1,150.9)	$1,108.4 ($1,101.1)
**Total cost of cardiac-specific medication**					
Total cost, sum	$15,415,350.6	$887,598	$1,176,184.6	$4,549,512.4	$6,613,295.1
Mean (SD) annual costs	$1,037.9 ($2,191.3)	$2,428.6 ($3,663.2)	$1,644.7 ($2,239.2)	$1,634.8 ($2,308.0)	$1,722.2 ($2,341.3)
Number of patients with positive[a] total cost, n (%)	12628 (83.6)	3633 (77.9)	3593 (79.5)	3833 (91.4)	4363 (93.6)
Mean (SD) annual costs in patients with positive[a] total cost	$1,241.1 ($2,343.0)	$3,116.4 ($3,882.8)	$2,066.2 ($2,329.9)	$1,785.8 ($2,355.6)	$1,840.2 ($2,374.9)
**Total cost of all included healthcare categories**					
Total cost, sum	$208,854,49.8	$13,713,103.8	$8,248,600.4	$19,906,150.3	$41,867,854.4
Mean (SD) annual costs	$14,062.6 ($26,451.5)	$38,301.8 ($165,342.7)	$12,406.7 ($68,321.7)	$8,682.4 ($29,292.3)	$14,343.7 ($53,900.8)
Number of patients with positive[a] total cost, n (%)	14,938 (98.9)	4,083 (87.6)	4,005 (88.7)	4,032 (96.2)	4,543 (97.4)
Mean (SD) annual costs in patients with positive[a] total cost	$14,215.1 ($26,553.8)	$43,733.3 ($176,006.6)	$13,983.5 ($72,382.3)	$9,018.4 ($29,803.1)	$14,719.4 ($54,551.7)

[a] Positive total cost defined as patients with ≥1 HCRU with an associated cost.

COPD, chronic obstructive pulmonary disease; ED, emergency department; GP, general practitioner; HCRU, healthcare resource utilisation; SD, standard deviation; SP, specialist physician.
